# A central role for dityrosine crosslinking of Amyloid-β in Alzheimer’s disease

**DOI:** 10.1186/2051-5960-1-83

**Published:** 2013-12-18

**Authors:** Youssra K Al-Hilaly, Thomas L Williams, Maris Stewart-Parker, Lenzie Ford, Eldhose Skaria, Michael Cole, William Grant Bucher, Kyle L Morris, Alaa Abdul Sada, Julian R Thorpe, Louise C Serpell

**Affiliations:** 1 School of Life Sciences, University of Sussex, Falmer BN1 9QG, UK; 2 College of Sciences, Chemistry department, Al-Mustansiriyah University, Baghdad, Iraq; 3 Physics Department, Drexel University, Philadelphia PA 19104, USA; 4 Current address: School of Life Sciences, Gibbet Hill Campus, University of Warwick, Coventry CV4 7AL, UK

**Keywords:** Amyloid, Oligomer, Aggregation, Alzheimer’s disease, Dityrosine, Oxidative stress, Electron microscopy

## Abstract

**Background:**

Alzheimer’s disease (AD) is characterized by the deposition of insoluble amyloid plaques in the neuropil composed of highly stable, self-assembled Amyloid-beta (Aβ) fibrils. Copper has been implicated to play a role in Alzheimer’s disease. Dimers of Aβ have been isolated from AD brain and have been shown to be neurotoxic.

**Results:**

We have investigated the formation of dityrosine cross-links in Aβ42 formed by covalent ortho-ortho coupling of two tyrosine residues under conditions of oxidative stress with elevated copper and shown that dityrosine can be formed in vitro in Aβ oligomers and fibrils and that these links further stabilize the fibrils. Dityrosine crosslinking was present in internalized Aβ in cell cultures treated with oligomeric Aβ42 using a specific antibody for dityrosine by immunogold labeling transmission electron microscopy. Results also revealed the prevalence of dityrosine crosslinks in amyloid plaques in brain tissue and in cerebrospinal fluid from AD patients.

**Conclusions:**

Aβ dimers may be stabilized by dityrosine crosslinking. These results indicate that dityrosine cross-links may play an important role in the pathogenesis of Alzheimer’s disease and can be generated by reactive oxygen species catalyzed by Cu^2+^ ions. The observation of increased Aβ and dityrosine in CSF from AD patients suggests that this could be used as a potential biomarker of oxidative stress in AD.

## Background

Amyloid fibrils are associated with a large number of diseases in which proteins or peptides abnormally assemble to form insoluble amyloid deposits in the tissues. The amyloid-β (Aβ) protein is an amyloidogenic peptide that is cleaved from the amyloid precursor protein (APP) [1]. The Aβ peptide is present in all individuals but in Alzheimer’s disease (AD) it abnormally assembles and deposits in amyloid plaques in the neuropil [1]. Amyloid fibrils are characterized by their significant insolubility and resistance to degradation [2]. Aβ is thought to play a central role in AD pathology since several familial AD mutations are related to changes within the Aβ peptide itself, or in the proteins affecting its production [3]. Aβ42 contains a tyrosine at position 10, located close to the fibrillogenic core of the peptide [4]. However, the tyrosine residue can oxidize to many different modification products, such as nitrotyrosine and dityrosine [5].

Redox-active metal ions, such as Cu^2+^ and Fe^3+^, have been suggested to play a role in the pathogenesis of many neurodegenerative disorders, including AD and Parkinson’s disease [6]. There is a large body of evidence pointing to the importance of interactions between redox-active metal ions and proteins in the pathogenesis of many diseases [7-9]. Two mechanisms have been suggested to explain the abnormalities of these interactions in neural tissue: (a) the aggregation of protein mediated by redox-active metal ions; and (b) metal catalyzed oxidation reactions (MCO) [10]. Metal-protein interactions could result in oxidative stress through generation of reactive-oxygen species (ROS), which in turn induces lipid peroxidation, protein oxidation, and DNA damage [11,12]. The consequences of protein oxidation are protein cross-links, amino acid side chain modifications, and protein fragmentation [11,13]. The oxidative modification of proteins by ROS has been classified in two groups; (a) Global oxidative modifications, which include oxidation of multiple residues within protein to form several products; (b) Specific oxidative modifications which are very specific in both the residue oxidized and the product generated, e.g. oxidation of tyrosine residue to give dityrosine [14].

High concentrations of copper (0.4 mM), zinc (1 mM), and iron (1 mM) have been found in amyloid plaques and have been implicated in the pathogenesis of AD [10,15]. Several studies have shown that Aβ is able to reduce copper and iron ions and to generate ROS [7,9,12,16]. The most common *in vivo* source of ROS is hydrogen peroxide (H_2_O_2_) breakdown according to the Fenton reaction, which is catalyzed by a metal-protein complex [7].

The formation of dityrosine cross-links is one of the oxidative modifications that have been implicated in mediating toxicity of Aβ through Aβ aggregation. Several studies [17-20] demonstrate that Aβ can undergo dityrosine formation via two common biochemical pathways. One of them is peroxidase-catalyzed cross-linked tyrosine and the second mechanism is metal-catalyzed oxidative tyrosyl radical formation. The mechanism of dityrosine cross-links involves tyrosyl radical formation, followed by radical isomerisation and then diradical reaction, and finally enolisation [21]. On the other hand, Smith et al. demonstrated that the generation of the Aβ toxic species is modulated by the concentration of Cu^2+^ ions and the ability to form intermolecular histidine bridges [22]. Interestingly dityrosine has been selected as a biomarker for oxidative stress of proteins due to its chemical stability, as it remains unchanged by exposure to oxygen and high pH [23]. Furthermore, it is highly resistant to acid hydrolysis and proteases [24,25].

Here we have explored the *in vitro* formation of dityrosine in Aβ42 using Cu^2+^ ions and H_2_O_2_ in both early oligomeric Aβ42 and preformed Aβ42 fibrils and examined the effect of the cross-linking on the structure of the fibrils and the assembly competence of the oligomers. We have shown that dityrosine formation is inducible in both soluble and fibrillar Aβ42 and suggest that the formation of crosslinks may stabilize assemblies. Neuroblastoma cell cultures treated with non-oxidized Aβ42 oligomers showed formation of Aβ assemblies containing dityrosine surrounding and sometimes internalized into the cells. Examination of samples from AD patients revealed the presence of dityrosine linkages within amyloid plaques and its colocalisation with Aβ in cerebrospinal fluid pointing to a physiological relevance of dityrosine crosslinking in AD, and highlighting the importance of oxidative stress in the disease.

## Methods

### Synthesis of a dityrosine standard

A dityrosine standard was synthesized according to modifications of established procedures [20,26]. 20 units of horse radish peroxidase (HRP) were added to a clear solution of (10 mM) *N*-acetyl-3,5-diiodo-L-tyrosine in 40 ml of (0.1 M) phosphate buffer pH 6.0 containing 10% of acetonitrile then mixed gently. Immediately, 0.48 ml of (1 M) H_2_O_2_ was added then the reaction mixture was stirred gently for 60 min at 24°C, quenched with 1.2 ml of (1 M) NaHSO_3_ and the pH of the mixture was adjusted to 7.5 with (1 M) NaOH. After stirring for 10 min the mixture was acidified to pH 3.0 with (2 M) KHSO_4_ and then extracted with ethyl acetate. The combined organic residues were concentrated under vacuum to give a tan residue, which was purified by flash chromatography to give 3,3′-diiodo-*N,N’-*diacetyl di-L-tyrosine. In turn, 3,3′-diiodo-*N,N’-*diacetyl di-L-tyrosine was hydrogenated in 50% methanol:acetic acid to give *N,N′-*diacetyl di-L-tyrosine, which was heated at reflux in a 1:1 mixture of tetrahydrofuran and concentrated HCl to give dityrosine. Gel filtration on sephadex G-10 was carried out in order to remove the salt and further purification was achieved using RP-HPLC. Finally, the purified dityrosine was dried using a freeze dryer (Edwards, England), and analyzed using a Bruker Daltonics APEX III 4.7 Tesla Fourier Transform ion cyclotron resonance mass spectrometer (FT-ICR-MS) with electrospray source and found to be *m/z* 361.1392 compared with calculated *m/z* 361.1394. NMR was carried out and the ^1^H NMR spectrum contained six proton signals with chemical shifts of: 7.30 ppm (H, d), 7.21 ppm (H, s), 7.07 ppm (H, d), 4.26 ppm (H, m), 3.35 ppm (H, dd), and 3,26 ppm (H’, dd). This spectrum is very similar to that published elsewhere [27-29].

### Preparation of fibrillar and oligomeric Aβ42

1,1,1,3,3,3-hexafluoro-2-propanol (HFIP) Aβ42 was purchased from rPeptide (Bogart, GA, USA). To remove preformed aggregates, the peptide was prepared as previously described [30] using HFIP >99.0%, followed by anhydrous DMSO (Fisher Sci.) and then buffer exchanged using a desalting column to remove the solvents. The concentration was determined using a molar extinction coefficient of 1490 M^-1^ cm^-1^ and the absorbance was measured at a wavelength of 280 nm using an Eppendorf Biophotometer (Eppendorf UK Ltd., Cambridge, UK). The resulting stock peptide concentrations of 90–130 μM were used immediately for early oligomeric Aβ42 experiments, or incubated for at least two weeks at room temperature (22°C) in order to generate Aβ42 fibrils (confirmed using TEM).

### Oxidation of fibrillar and early oligomeric Aβ42

Stock solutions of soluble or fibrillar Aβ42 were diluted in (a) water or (b) 50 mM phosphate buffer pH 7.4 to obtain 20 μM Aβ42 as a final concentration and incubated with or without (20 μM) Cu^2+^ and (0.5 mM) H_2_O_2_ at 37°C with agitation in a shaking incubator for three days. The oxidation reaction was quenched using a final concentration of 250 μM EDTA.

### Fluorescence spectroscopy

The fibrils were resuspended by agitation. Fluorescence spectra were recorded over time. Fluorescence measurements were carried out on a Varian Cary Eclipse fluorimeter (Varian Ltd., Oxford, UK) using a 1 cm path length quartz cuvette (Starna, Essex, UK), and dityrosine fluorescence was monitored using an excitation wavelength of 320 nm. Dityrosine emission was monitored between 340 and 500 nm, with maximum fluorescence intensity at around 400–420 nm at a controlled temperature of 21°C. Tyrosine fluorescence signal was monitored using an excitation wavelength of 280 nm and emission wavelength of 305 nm. Excitation and emission slits were both set to 10 nm, and the scan rate was set to 300 nm/min with 2.5 nm data intervals and an averaging time of 0.5 s. The photomultiplier tube detector voltage was set at 500 V. To detect dityrosine fluorescence at early time points, 130 μl of the reaction mixture was removed and EDTA was added to final concentration of 250 μM.

### Sample preparation for LC-ESIMS/MS analysis

Oxidized Aβ42 fibrils prepared in water were lyophilized using a Modulyo 4 K Freeze Dryer (Edwards, Crawley, England), and then hydrolysed using evacuated sealed tubes under acidic conditions of (6 M) HCl, 10% TFA, and 1% phenol at 110°C for 48 h. The resulting hydrolysate was then dried under nitrogen gas, dissolved in 100 μl of 0.1% formic acid in water and then filtered using a Millipore 0.22 μm filter into a 0.2 ml tube.

### Detection of dityrosine by LC-ESIMS/MS

20 μl of oxidized Aβ42 fibril hydrolysate was injected on to a Phenomenex Gemini 3u C_6_-phenyl 110 (150 mm × 4.6 mm, 3 micron) column using a High performance liquid chromatography (HPLC) system (Waters Alliance 2695, Ireland) coupled to the mass spectrometer (MicroMass Quattro Premier, Waters, Ireland) operated in the multiple reaction-monitoring (MRM) mode with positive electrospray ionisation (ESI). The solvents for the mobile phase were A: 0.1% formic acid in water; and solvent B: 0.1% formic acid in acetonitrile. The gradients were as follows: t = 0 min, 0% B; t = 1 min, 0% B; t = 15 min, 100% B; t = 20 min, 100% B; t = 25 min, 0% B; t = 30 min, 0% B, and the flow rate was 200 μl/min. Mass spectrometric detection was performed by positive electrospray ionisation (ESI) tandem mass spectrometry on a triple quadrupole mass spectrometer (MicroMass Quattro Premier, Waters, Ireland). The conditions for the mass spectrometer were as follows; electrospray ionization spray voltage 3.5 kV, the cone voltage 35 V, the source temperature at 100°C, whereas the desolvation temperature was 400°C. Argon was used as the collision gas at 5.95 e^-^003 mbar at 26 ev collision energy.

### Thioflavin T fluorescence assay

The fibril formation of Aβ42 was monitored using ThT fluorescence. A 0.2 μm filtered (3.14 mM) aqueous ThT stock solution was prepared and stored frozen at −20°C in 1–10 μl aliquots until required. ThT was added to a 10 μM Aβ42 sample (50 mM phosphate buffer pH 7.4) to a final concentration of 20 μM, gently vortexed, and allowed to bind for 3 minutes before a reading was taken. Using a microvolume cuvette of 1 cm path length, ThT fluorescence was measured using a Varian Cary Eclipse fluorimeter (Varian, Oxford, UK) with excitation wavelength of 450 nm. The emission spectrum was recorded between 460–600 nm at 21°C. Phosphate buffer baselines were subtracted from the data. Excitation and emission slits were set to 5 nm and 10 nm respectively. The scan rate was 600 nm/min with 1 nm data intervals and an averaging time of 0.1 s. The voltage on the photomultiplier tube was set to high (800 v) and experiments were carried out in triplicate to confirm trends.

### Negative stain TEM

Four microliter aliquots of Aβ42 samples were placed onto Formvar/carbon coated 400-mesh copper grids (Agar Scientific, Essex, UK) for 1 min, and the excess was removed using filter paper. Subsequently the grid was washed using 4 μl of Milli-Q water filtered with 0.22 μm filter and blotted dry, then negatively stained twice with 4 μl of filtered 2% (w/v) uranyl acetate for 1 min and blotted dry. The grid was allowed to air-dry before examination on a Hitachi 7100 transmission electron microscope (Hitachi, Germany) fitted with a Gatan Ultrascan 1000 CCD camera (Gatan, Abingdon, UK) at an operating voltage of 100 kV.

### Dityrosine cross-linked Aβ42 fibril stability

Aβ42 was assembled under oxidizing and non-oxidizing conditions and stored at −80°C for more than one year and the dityrosine content was assessed using fluorescence with an excitation wavelength of 320 nm as described above. Both oxidized and non-oxidized Aβ42 fibrils were centrifuged for 30 min at 16,000 RCF at 4°C, and then the soluble Aβ42 concentration in the supernatant was measured using absorbance at 280 nm (as described for peptide preparation). TEM grids were prepared to examine the Aβ42 fibrils in the pellet. The oxidized and non-oxidized fibrils were then dissolved in 80% v/v formic acid with agitation. The resulting solution was centrifuged using the same conditions above, and again the dissolved Aβ42 concentration was determined. TEM grids for the pellet were prepared to examine the morphology of the dissolved fibrils and to compare their density.

### SDS gel electrophoresis

Aβ was incubated under oxidizing conditions as before for 10 mins and then quenched using EDTA. Oxidized and non-oxidized Aβ peptides were separated by SDS-PAGE and analyzed by densitometry. Samples were heated to 85°C for 5 min and then 5 μl added to each well of a Novex® 1.0 mm 10-20% Tricine gel, (Life Technologies Ltd, Paisley, UK). Oxidized and nonoxidized samples were run on the XCell SureLock® Mini-Cell with a PowerEase® 500 power supply (Life Technologies Ltd, Paisley, UK). A voltage of 125 V, an expected start current of 80 mA/gel and an expected end current of 40 mA/gel was applied. The gel was stained using SilverQuest™ Silver staining kit (Life Technologies Ltd, Paisley, UK) following the manufacturers protocol provided. Briefly, the gel was fixed for 60 min in 40% ethanol, 10% acetic acid, and 50% milliQ water, then rinsed in 30% ethanol (10 min), incubated in sensitizer solution (10 min), rinsed in 30% ethanol (10 min), washed with milliQ water (10 min), incubated in silver stain (15 min), and washed again with milliQ water (60 sec). The gel was then incubated in developer (5–10 min). Finally, the stopper was directly added to the stained gel (10 min) and subsequently washed with milliQ water (10 min). The gel was scanned at 8 bit with 600 dpi resolution. The densitometry was calculated using ImageJ.

### Immunogold labeling, negative stain TEM (for fibrils and cerebrospinal fluid)

A modified phosphate-buffered saline, pH 8.2, containing 1% BSA, 500 μl/l Tween-20, 10 mM Na EDTA, and 0.2 g/l NaN_3_ (henceforward termed PBS+), was used throughout all the following procedures for all dilutions of antibodies and secondary gold probes. Aβ42 fibrils were oxidized and assembled as described in oxidation of fibrillar and early oligomeric Aβ42 and immunogold-labeled ‘on grid’ for dityrosine according to established methods. A monoclonal anti-dityrosine antibody was purchased from Japan Institute for the Control of Aging (JaICA) Shizuoka, Japan (cat. no. MDT-020P). The antibody has been fully characterised and shown to be highly specific and does not show any cross-reactivity with other tyrosine derivatives such as nitrotyrosine, chlorotyrosine [31]. The antibody will detect any protein containing dityrosine. Briefly, 4 μl aliquots of the oxidized Aβ42 fibrils were pipetted onto Formvar/carbon coated 400 mesh copper TEM support grids (Agar Scientific, Essex, UK), left for 1 min, the excess was removed with filter paper, and then blocked in normal goat serum (1:10 in PBS+) for 15 min. Grids were then incubated with (10 μg/ml IgG) mouse dityrosine monoclonal antibody (JaICA, Shizuoka, Japan) for 2 h at room temperature, rinsed in 3×2 min PBS+, and then immunolabeled in a 10 nm gold particle-conjugated goat anti-mouse IgG secondary probe (GaM10 British BioCell International, Cardiff, UK; 1:10 dilution) for 1 h at room temperature. After 5×2 min PBS + and 5×2 min distilled water rinses, the grids were negatively stained as described in negative stain TEM.

Cerebrospinal fluid (CSF) samples from AD patients and control age matched subjects were obtained from the London Neurodegenerative Diseases Brain Bank. CSF was removed according to Local Ethics Committee guidelines, and informed consent for brain donation was obtained from the next of kin (see Table 1). The samples were stored at −80°C in a locked freezer until needed. When required, they were diluted with Milli-Q water (1:3). A rabbit antibody raised against N-terminus of Aβ42 (1–6) (AB5078P, Chemicon, Temecula, CA, USA) was used to detect Aβ in the following experiments. This antibody has been shown not to cross react with APP [32]. Diluted CSF samples were double-immunogold-labeled for dityrosine and Aβ42 as described above, except grids were incubated in a mixture of (10 μg/ml IgG) anti Aβ42 rabbit polyclonal antibody AB5078P (Chemicon, Temecula, CA, USA) and (10 μg/ml IgG) mouse monoclonal dityrosine antibody (Japan Institute for the Control of Aging JaICA, Shizuoka, Japan). A mixture of 5 nm gold particle-conjugated goat anti-rabbit IgG (GaR5 British BioCell International, Cardiff, UK) and 15 nm gold particle-conjugated GaM (British BioCell International, Cardiff, UK; 1:10 dilution) was used for the secondary gold probe.

**Table 1:** Demographic details of cases from which cerebrospinal fluid were obtained

**Case**	**Age**	**Sex**	**Pathological diagnosis**
AD 1	68	M	Alzheimer’s disease HP-tau stage 6 with mild to moderate amyloid angiopathy
AD 2	93	F	Alzheimer’s disease HP-tau stage 6 with moderate amyloid angiopathy
AD 3	91	M	Alzheimer’s disease HP-tau stage 6 with moderate amyloid angiopathy
Normal 1	89	F	Control case but with hypoxic-type changes and amyloid angiopathy
Normal 2	92	F	Control-some amyloid angiopathy

### Immunogold Labeling TEM of sections

AD and aged matched control brain from middle frontal gyrus tissues were obtained from London Neurodegenerative Diseases Brain Bank. Tissue was removed according to Local Ethics Committee guidelines, and informed consent for brain donation was obtained from the next of kin (see Table 2) and stored at −80°C until required. SH-SY5Y cells were incubated for 24 hours with a final concentration of freshly prepared 10 μM Aβ42 and controls cells were administered with buffer only (prepared as described in Soura *et al*., 2012 [33]). SH-SY5Y cells and brain tissues were prepared for immunogold labeling TEM by minimal, cold fixation and embedding protocols, as previously described in Soura *et al.,* (2012) [33] and Thorpe *et al.,* (2001) [34], respectively. Immunogold labeling was performed using an established methodology [35], with PBS + buffer being used for all dilutions of immunoreagents and for rinsing. Thin sections were collected upon TEM support grids, then incubated with normal goat serum (1:10 dilution) for 30 min at room temperature to block non-specific secondary antibody binding. In turn, grids were labeled with (10 μg/ml IgG) anti-dityrosine mouse monoclonal antibody (Japan Institute for the Control of Aging JaICA, Shizuoka, Japan) or double-labeled using a mixture of (10 μg/ml IgG) anti-Aβ42 rabbit polyclonal antibody AB5078P (Chemicon, Temecula, CA, USA) and (10 μg/ml IgG) anti-dityrosine mouse monoclonal antibody and incubated overnight at 4°C. After 3×2 min PBS + rinses, sections were then immunolabeled with GaM10 or a mixture of GaR5 and GaM10 secondary probes (both 1:10 dilution), respectively, for 1 h at room temperature. After 3×10 min PBS + and 4×5 min distilled water rinses, the grids were post-stained in 0.22 μm-filtered 0.5% (w/v) aqueous uranyl acetate for 1 h. Labelling controls were performed using serial sections and using the identical procedure and IgG concentrations, with an irrelevant antibody to hair cell antigen (MAb10) (Richardson et al., personal communication).

**Table 2:** Demographic details of cases from which middle frontal gyrus tissues were obtained

**Case**	**Age**	**Sex**	**Pathological diagnosis**
Normal 1	89	F	Control case but with Hypoxic-type changes and amyloid angiopathy
Normal 3	80	F	Control-minimal ageing changes
AD 1	68	M	Alzheimer’s disease HP-tau stage 6 with mild to moderate amyloid angiopathy
AD 2	93	F	Alzheimer’s disease HP-tau stage 6 with moderate amyloid angiopathy
AD 3	91	M	Alzheimer’s disease HP-tau stage 6 with moderate amyloid angiopathy
AD 4	86	F	Alzheimer’s disease HP-tau stage 6 with mild amyloid angiopathy
AD 5	77	F	Alzheimer’s disease -modified Braak BNE stage 5

The grids were examined on a Hitachi 7100 TEM (Hitachi, Germany) fitted with a Gatan Ultrascan 1000 CCD camera (Gatan, Abingdon, UK), and operating with a voltage of 100 kV.

### Analysis of immunogold labeled sections

Counting and size analyses of immunogold particles were performed using in house software written in Matlab. Briefly, raw digital electron micrograph images files were uploaded and circular particles detected using inbuilt Matlab circular Hough transforms functions. The detection range diameter was calculated from user input and conversion to pixel size values read from the image header. 15 or 10 nm gold particles were distinguished from 5 nm particles based on a user-selected threshold of 7 nm.

## Results

### *In vitro* oxidation of Aβ42 resulting in the formation of dityrosine cross-links

**Figure 1:**
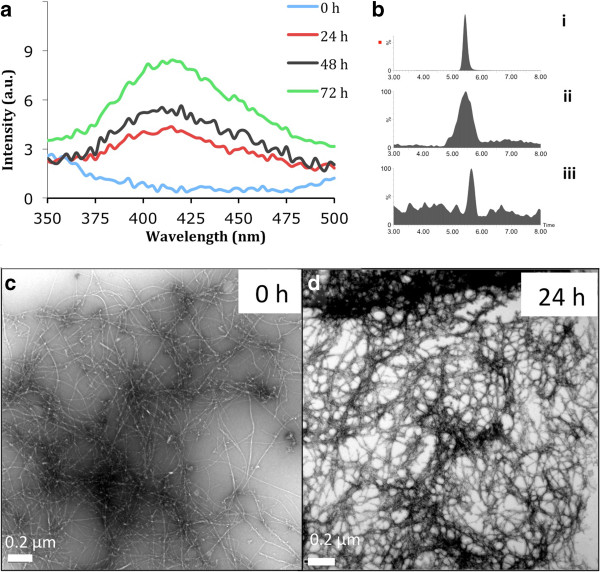
**Formation of dityrosine crosslinks in Aβ42 fibrils.** Preformed Aβ42 fibrils (20 μM) were incubated in the presence of Cu^2+^/H_2_O_2_ for 72 hours and the appearance of dityrosine detected using fluorescence **(a)**. **b)** The dityrosine content was confirmed using LC-ESIMS/MS and this shown with relative abundance on y-axis. i) LC-ESIMS/MS from authentic synthetic dityrosine, ii) hydrolysate from oxidized preformed Aβ42 fibrils, iii) hydrolysate from Aβ42 fibrils formed under oxidation conditions for three days (the fibrils were obtained from incubation of soluble Aβ42 with Cu^2+^/H_2_O_2_ in water at 37°C and agitation) **(c, d)** Electron micrographs showing the morphology of fibrils prior to oxidation **(c)** and following 24 hour oxidation **(d)** (diameters approximately 100–150 Å).

Aβ42 amyloid fibrils were preformed in water and incubated for up to three days in the presence of Cu^2+^ and H_2_0_2_ in phosphate buffer pH 7.4 to induce dityrosine cross-link formation by oxidation. The fibrils were examined using fluorescence spectrometry and an increasing signal at 400–420 nm corresponding to dityrosine [36] was observed with time of oxidation (Figure 1a). To further confirm the presence of dityrosine within the oxidized Aβ42 fibrils, the fibrils (prepared in water) were hydrolyzed using 6 M HCl, 10% TFA, and 1% phenol for 48 h and examined using LC-ESIMS/MS (Figure 1b). The dityrosine content was identified using transition reactions ions 361.1/315 and a retention time of 5.5 min (Figure 1bii), consistent with that of a dityrosine standard (Figure 1bi). Negative stain transmission electron microscopy (TEM) was used to compare oxidized fibrils with non-oxidized fibrils to evaluate any potential morphological changes to the samples and revealed short, clumped networks of fibrils in the oxidized sample after 24 hours (Figure 1d) compared to long, straight, and well dispersed fibrils with diameter of 9.32 nm (SD ±2.1 n = 6) observed in the sample without oxidation and incubated in phosphate buffer only (Figure 1c). This may suggest that the oxidized fibrils are cross-linked to form a network and this is consistent with tyrosine residue 10 being exposed on the surface of the fibrils and available for dityrosine crosslinking between either protofilaments or perhaps individually crossing fibrils. Structures for Aβ42 and Aβ 40 fibrils show the tyrosine residue at position 10 at the end of β-sheet core [4,37] showing that they may be available to be involved in lateral association.

**Figure 2:**
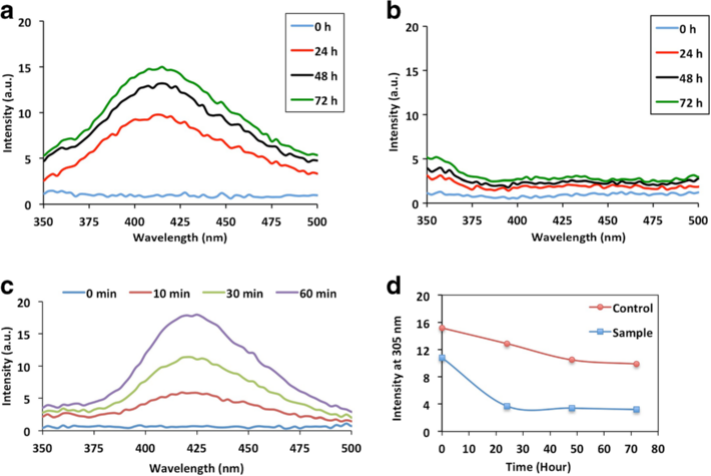
**Monitoring dityrosine and tyrosine fluorescence during Aβ42 assembly.** Freshly prepared Aβ42 (20 μM) was incubated in the presence of Cu^2+^/H_2_O_2_ and monitored by fluorescence over three days **(a)** and compared to Aβ42 alone **(b)**. Dityrosine fluorescence was monitored (Ex. 320 nm, Em. 410–420 nm) and a) oxidized Aβ42 shows a strong signal at 24 hours incubation compared to no signal in **b)** control Aβ42 sample. The development of the dityrosine signal was monitored at earlier time points **(c)** using the addition of EDTA and the spectrum shows a signal at 420 nm following only 10 mins incubation. Tyrosine fluorescence (Ex. 280 nm, Em. 305 nm) was used to follow assembly with time **(d)**. The intensity at 305 nm is plotted against time. Both conditions show a decrease in fluorescence signal over time for tyrosine, but oxidized Aβ42 shows a significant reduction after 24 hours, compared to a slow reduction in tyrosine fluorescence that accompanies assembly for control Aβ42.

Many recent studies have demonstrated that soluble Aβ oligomers, rather than Aβ fibrils, are the most toxic species and disruptive to physiological processes involved in learning and memory in AD [33,38,39]. Dimeric species have been isolated from AD brains [40] and these have also been shown to be toxic [41]. Oligomers formed by synthetic dityrosine crosslinked dimers have been shown to be more toxic than corresponding monomeric peptide [42]. In order to investigate whether dityrosine crosslinks can be formed by soluble Aβ42, freshly solubilized Aβ42 was incubated up to three days in the presence and absence of Cu^2+^ and H_2_0_2_ in phosphate buffer at pH 7.4 to induce crosslinking as well as to follow fibrillogenesis. Dityrosine formation was monitored using fluorescence excitation over three days (Figure 2a,b) and confirmed by LC-ESIMS/MS (Figure 1biii). The resulting assemblies were also assessed using TEM, Thioflavin T (ThT) fluorescence and SDS-PAGE.

**Figure 3:**
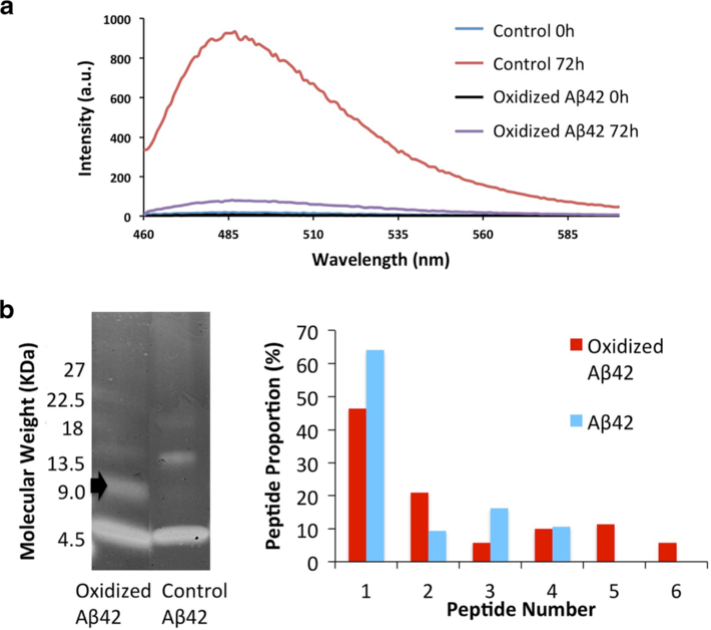
**Thioflavine T fluorescence and SDS PAGE of oxidized and control Aβ42. a)** ThT fluorescence spectra following fibril formation from Aβ42 (20 μM) in the presence or absence of Cu^2+^ and H_2_O_2_ for 72 hours. The spectra for oxidized and control samples show an increased intensity over the incubation time and show increased ThT signal for non-oxidized compared to oxidized samples. **b)** SDS PAGE showing separation of Aβ42. Oxidized Aβ42 (left column) runs as monomer and dimer (approx. 9 kDa, black arrow), whilst non oxidized, control Aβ42 (right column) shows bands corresponding to monomer and trimer as previously observed [44]. The trimer is thought to be induced by SDS [44]. Densitometry confirms that monomer is the strongest band following by dimer for oxidized but not control fibrils. This reveals that the dimer is enriched under oxidation conditions.

**Figure 4:**
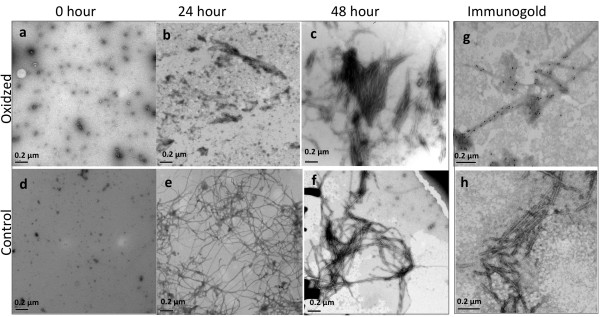
**Transmission electron microscopy images of freshly formed Aβ42 fibrils.** 20 μM Aβ42 (pH 7.4) was incubated in the presence of Cu^2+^/H_2_O_2_**(a, b, c)** and compared to 20 μM Aβ42 in phosphate buffer alone (pH 7.4) **(d, e, f)**. Assembly was monitored by electron microscopy after incubation for (a and d) zero hours, (b and e) 24 hours and (c and f) 48 hours. Both oxidized and control Aβ42 samples show oligomeric species at zero hour **(a, d)** and fibrils following 48 hour incubation **(c, g)**. However, at 24 hours (b and f) fibrils were observed in non-oxidized conditions **(f)**, but no fibrils were observed in oxidised conditions **(b)**. The presence of dityrosine was detected using immunogold labeling using a dityrosine specific antibody that labeled oxidized fibrils **(g)** but not control fibrils **(h)**.

Fluorescence spectra revealed an increasing dityrosine fluorescence signal with time in the Aβ42 sample incubated in the presence of Cu^2+^ and H_2_0_2_ (Figure 2a), whilst no such signal was observed in the control sample (Figure 2b). To examine how quickly dityrosine crosslinking develops, the oxidized sample was monitored using fluorescence over one hour (Figure 2c) revealing the presence of dityrosine after only 10 minutes incubation. In contrast, at an excitation wavelength of 280 nm, the tyrosine fluorescence signal at 305 nm was observed to decrease over the incubation time of three days (Figure 2d). A gradual decrease in tyrosine intensity was observed for control fibrils (Figure 2d) and this decrease is thought to correspond to involvement of tyrosine residues in Aβ42 assembly [43] or could be due to loss of signal as the fibrils elongate and precipitate. Tyrosine fluorescence spectra revealed a very significant loss of tyrosine signal concurrent with the increase in dityrosine signal in the oxidized sample following 24 hours incubation (Figure 2d) at the same time as a strong dityrosine signal is observed. At time zero, the intensity at 305 nm is lower for the oxidized sample compared to control and this is arises from quenching of the signal due to binding of the Cu^2+^ to tyrosine. Dityrosine formation appears to occur early (after only 10 mins) in the assembly process consistent with dityrosine coupling being present in early oligomeric species. A ThT fluorescence assay showed an increased signal following 72 hour incubation of both control and oxidized Aβ42 confirming formation of fibrils in both samples (Figure 3a). However, the intensity of the signal from control fibrils is significantly higher than for oxidized fibrils. This may arise from the effect of differences in the buffer conditions on ThT signal or possibly due to the increased association of fibrils formed by oxidized Aβ42 revealed by electron microscopy (Figure 4). Following only 10 minutes incubation, SDS gel electrophoresis separation of oxidized Aβ42 reveals the presence of bands at molecular weights corresponding to dimer to hexamer (Figure 3b), similar to those shown by oxidation using PICUP [44], which supports the view that small Aβ assemblies can be stabilized by oxidation to dityrosine. In comparison, Aβ incubated without oxidation runs as a monomer and a trimer, but no significant dimer is observed (Figure 3b). These SDS stable oligomeric species appear to evolve further and we observe fibrils following 48 incubation (Figure 4) suggesting that the formation of the cross-linked dimer could be on pathway for fibril assembly.

To gain further insight into the morphological changes to Aβ42 fibrils induced by oxidation, samples were examined using negative stain TEM (Figure 4). Aβ42 immediately following preparation (zero hour) showed small globular structures consistent with oligomeric species (5–30 nm) (Figure 4a and 4d). Under oxidation conditions the freshly dissolved Aβ42 oligomers again appeared to have a spherical appearance (5–25 nm) (Figure 4a) and these developed into larger, non-fibrillar structures after 24 hours (Figure 4b) and then into clustered fibrils after 48 hours (Figure 4c). After incubation for 24 hours in phosphate buffer only, fibrils were observed with both flat, striated ribbons, and twisted morphologies (Figure 4e) and these developed further at 48 hours (Figure 4f). TEM did not reveal any obvious differences in fibril density between Aβ incubated under oxidizing and non-oxidizing conditions, although the oxidized sample appears to show shorter, more self-associated fibrils, which may be consistent with some interfibrillar crosslinking. Fibrils were observed following 24 hour incubation in control samples, but not until 48 hours for the oxidized Aβ42.

In order to examine whether dityrosine crosslinks can be detected within the amyloid fibrils and to show the dityrosine distribution, TEM immunogold labeling using a dityrosine specific monoclonal antibody was performed. Figure 4e shows anti-dityrosine labeling on Aβ42 fibrils revealing gold distributed close to the fibrils grown in an oxidizing environment. Control Aβ42 did not label with the dityrosine antibody (Figure 4f). Very strong evidence of dityrosine identity was provided using these specific monoclonal dityrosine antibodies and these clear differences between oxidized and control also provides evidence for the specificity of the antibody. We are not able to rule out formation of trityrosine, but this would be expected to further strengthen the fibrils. Mass-spectrometry data (Figure 1b) shows strong evidence for the presence of dityrosine. These *in vitro* findings suggest that conditions similar to oxidative stress can promote the formation of dityrosine cross-links in self-assembling Aβ42 samples resulting in a high density within the fibrils. Interestingly, no fibrils were observed in the oxidized sample at 24 hours by TEM (data not shown), although there is a very strong signal by dityrosine fluorescence (Figure 2a & c). This supports the view that dityrosine cross-links form early in the assembly process.

**Figure 5:**
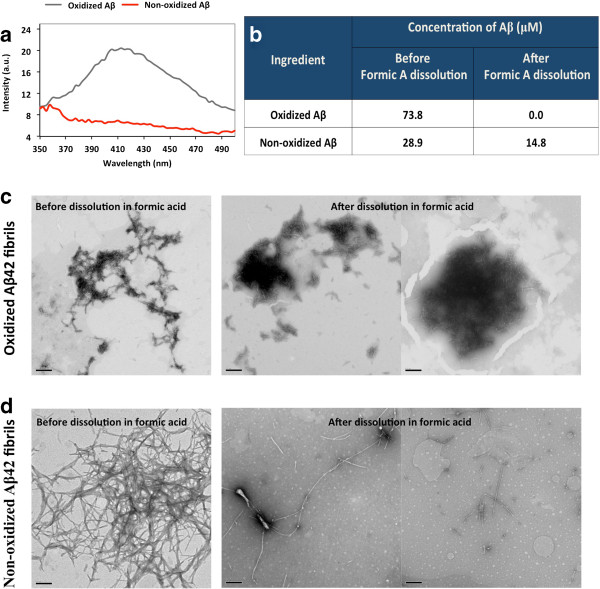
**The stability of crosslinked fibrils. a)** Oxidized and non-oxidized Aβ42 fibrils were examined using dityrosine fluorescence after prolonged incubation at −80°C showing a strong intensity signal at 420 nm for oxidized but not non-oxidized fibrils. **b)** Formic acid was used to dissolve the fibrils and the concentration of Aβ42 in solution was compared before and after formic acid dissolution for oxidized and non-oxidized fibrils. **c)** Electron micrographs of oxidized fibrils before and after formic acid treatment showing that the dityrosine crosslinked fibrils are resistant to formic acid. **d)** Electron micrographs of non-oxidized fibrils before and after formic acid treatment showing the fibrils are susceptible to damage by formic acid. Scale bars represent 0.2 μm.

Dityrosine crosslinking may lend further stability to the already stable amyloid fibrils formed. To investigate this possibility, we compared the stability of oxidized and non-oxidized Aβ42 amyloid fibrils. Fibrils were stored at −80°C for over one year. Following thawing, dityrosine content was assessed using fluorescence and oxidized fibrils were shown to still contain dityrosine crosslinks after prolonged freezing, whilst no dityrosine fluorescence was detected for non-oxidized, frozen fibrils (Figure 5a). Both sets of fibrils were examined by electron microscopy, and both contained fibrils (Figure 5c and d). Comparison of soluble Aβ42 concentration in the supernatants indicated that there was more soluble Aβ in the oxidized samples (Figure 5b). The fibril pellets were treated with formic acid to dissolve fibrils and the amount of peptide released into the supernatant was compared between the oxidized and non-oxidized samples (Figure 5b). The concentration of Aβ found in the supernatant following formic acid treatment was very significantly lower in oxidized sample compared to the non-oxidized sample (0 versus 14.8 μM), suggesting that the oxidized fibrils were resistant to dissolution with the acid (Figure 5b). Electron microscopy revealed that the oxidized fibrils were shortened, but remained at a broadly similar concentration when compared to amounts prior to oxidation. Those that had not been oxidized were narrower and much more difficult to find on the grid (Figure 5c and d). These results seem to support the view that the dityrosine crosslinking strengthens the fibrils and these fibrils become more resistant to acid dissolution following oxidation.

### Aβ42 is internalized into neuroblastoma cells and becomes dityrosine crosslinked

**Figure 6:**
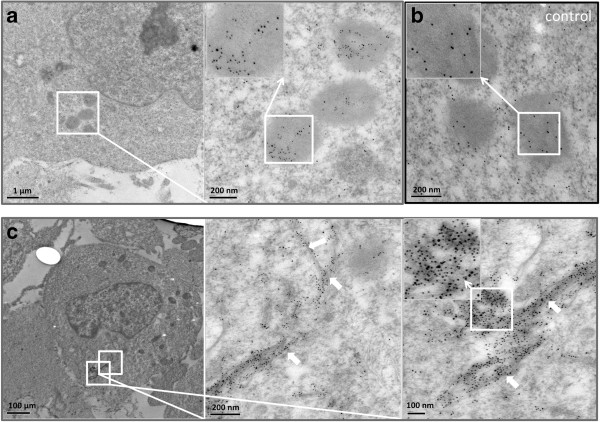
**Immunogold labeling TEM showing neuroblastoma cells treated with 10 μM oligomeric Aβ. a)** The images reveal dityrosine (10 nm) and Aβ42 (5 nm) labeling within the lysosomes of treated cells. **b)** low level dityrosine labeling was observed within lysosomes in vehicle treated, control cells, but no Aβ labeling was observed. **c)** Cells were observed containing fibrillar Aβ labeled for both Aβ (5 nm) and dityrosine (10 nm). White arrows are used to highlight the fibrillar material. Inserts show magnified images for further clarity.

**Figure 7:**
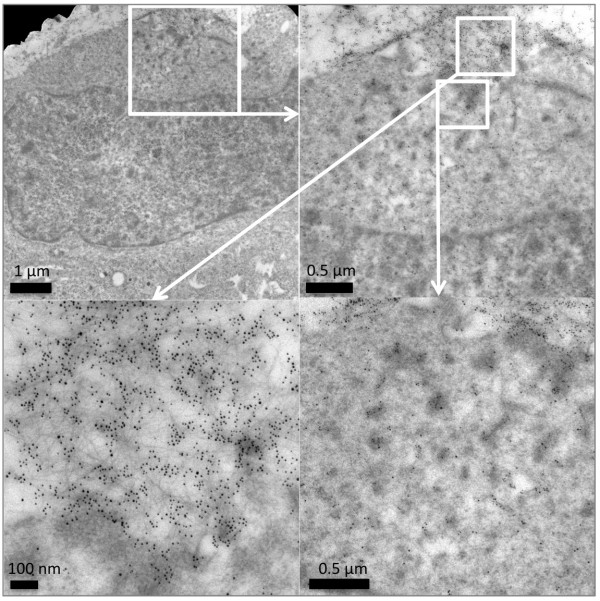
**Electron micrographs of sections of Aβ42 treated neuroblastoma cells showing immunogold labeling of Aβ (5 nm) and dityrosine (10 nm).** The images reveal colabeled fibrils outside and also inside the cells and highlight internalization at the plasma membrane (top left panel). The figure shows magnified images in the bottom two panels for extra clarity.

In previous work, we have shown that freshly prepared Aβ42 can be internalized into SH-SY5Y neuroblastoma cells and accumulates over 24 hours in lysosomal compartments [33]. We were interested to investigate whether the Aβ42 administered to these cells formed dityrosine crosslinks during incubation in contact with neuroblastoma cells. Cells were treated in an identical way to previous experiments [33], administered with a final concentration of freshly solubilized 10 μM Aβ42 and incubated for 24 hours. Sectioned cells were co-labeled with the mouse monoclonal dityrosine antibodies and rabbit polyclonal Aβ antibodies for Aβ42 fibrils. Previously, a monoclonal, conformational specific antibody was used to detect Aβ [33]. For the purpose of this study, secondary antibodies were 5 nm gold conjugated goat anti-rabbit IgG and 10 nm gold conjugated goat anti-mouse IgG, allowing the identification of both Aβ (5 nm) and dityrosine (10 nm) respectively in the sectioned cells and the opportunity to detect whether they co-localized. TEM of sectioned neuroblastoma cells treated with Aβ42 confirms the appearance of internalized Aβ42 concentrated in lysosomal regions (Figure 6a), as shown previously [33]. Close examination also showed that dityrosine and Aβ42 were co-localized together inside the lysosomes (Figure 6a). Examination of control cells showed some labeling of dityrosine within lysosomes, but no co-localization with Aβ labeling (Figure 6b). Further investigation revealed the presence of fibrillar Aβ42 around and within the cells, where the fibrils exhibited clear co-localization of dityrosine and Aβ (Figure 6c). This suggests that Aβ is cross-linked both inside and outside of the neuroblastoma cells. Occasionally extracellular Aβ was observed to be entering the cells at the plasma membrane (Figure 7) and again these showed co-localization of Aβ and dityrosine labeling. Taken together, these results suggest that the externally administered Aβ42 becomes oxidized during incubation in the cellular environment and that dityrosine crosslinked oligomers (and possibly fibrils) can be internalized into lysosomes.

### The presence of dityrosine within plaques in AD brain

**Figure 8:**
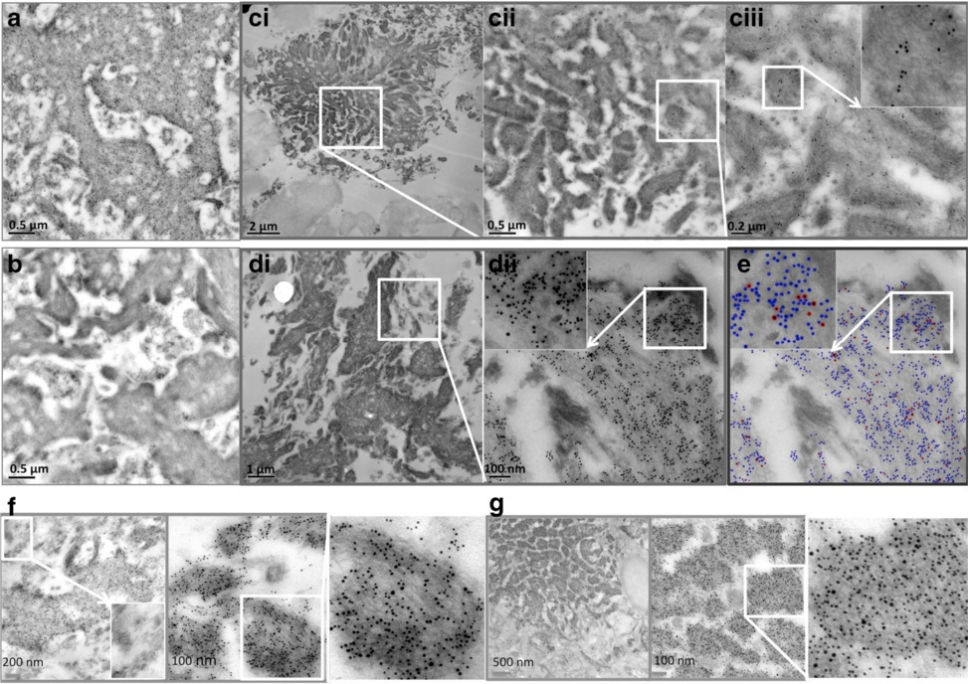
**Immunogold labeling TEM within amyloid plaques from AD brains. a)** Shows double labeling using the dityrosine antibody and anti-Aβ antibody on an AD brain section and reveals very dense labeling of the amyloid plaques. This is compared to a serial section **b)** Showing single labelling using an irrelevant antibody against hair cell antigen (HCA) to show the specificity of the dityrosine labeling on plaques. This image shows virtually no gold labeling. **c)** Shows single labeling using the dityrosine antibody to reveal very clear labeling at increasing magnification (ci, cii and ciii) showing that the dityrosine labels fibrils in the plaques. **d)** Double labeling with anti-Aβ (5 nm) and anti-dityrosine (10 nm) confirms colocalisation of dityrosine with Aβ within amyloid plaques at increasing magnifications (di and dii). **e)** Shows the 10 nm labeling for dityrosine highlighted in red and 5 nm labeling for Aβ in blue. Inserts show magnified images for further clarity. **f)** and **g)** show additional examples of co-immunogold labeling in brain from additional patients.

We have established that Aβ42 is able to undergo oxidation leading to formation of dityrosine crosslinks *in vitro* and in a cellular environment. To determine the possible physiological relevance of dityrosine formation in the Aβ accumulation in AD brain, immunogold labeling TEM was carried out to detect any co-localization of Aβ and dityrosine in AD brain and compared to age matched control brain. Previously dityrosine has been detected and quantified in AD brain [5]. Using HPLC with electrochemical array detector (HPLC-ECD), dityrosine was quantified in four regions of the AD brain [5]. It was reported that dityrosine levels were elevated significantly in the hippocampus and neocortical regions of the AD brain. However, no previous study has successfully detected dityrosine in plaques, and no cellular localization study of dityrosine in human brain has been reported before. Immunogold labeling using anti-dityrosine antibody was performed on brain sections taken from AD patients and control subjects (see Table 1), which revealed a high density of dityrosine within amyloid plaques in AD brain sections (Figure 8a and c). A control was performed using an irrelevant antibody (to hair cell antigen) at identical an IgG concentration and showed virtually no labeling (<<1 gold particles/micron) (Figure 8b) compared to the serial section showing dityrosine/Aβ labeling of the same plaque in Figure 8a (showing 100 gold particles/micron^2^). Figure 8c shows images revealing the clear labeling of the fibrillar component of the amyloid plaques with the dityrosine antibody (Figure 8ciii), indicating that the fibrils themselves contain dityrosine. Double immunogold labeling TEM was also used to confirm that the amyloid plaques labeled with the anti-Aβ antibody as well as anti-dityrosine (Figure 8di and diii) and this is highlighted by processing of the images to show different sized labels in red and blue in Figure 8e. The numbers of large (dityrosine) and small (Aβ) gold particles were compared between the amyloid plaque area and areas around the plaques and showed a level of 100 and 90 gold particles/micron^2^ for dityrosine and Aβ respectively over plaque areas. In comparison, the non-plaque areas showed only 2 gold particles/micron^2^ for both labels. Two additional AD patient brains were also examined and these are shown in Figure 8f and g and show very high density labeling over plaques. These results show the high density, specific localization of dityrosine within plaques (Figure 8) and strong evidence for colocalisation with Aβ, suggesting a potentially important role for dityrosine in amyloid accumulation in amyloid plaques.

### Dityrosine as a potential biomarker of oxidative stress in AD

Given our results showing the colocalisation of antibodies against dityrosine and Aβ over amyloid fibrils deposited in Alzheimer’s brains and images showing that neuroblastoma cells incubated with Aβ42 show dityrosine-Aβ colocalisation over fibrillar material and in lysosomes, we were interested to see if dityrosine crosslinked Aβ could be identified in cerebrospinal fluid (CSF) and could therefore be a useful biomarker for Alzheimer’s disease.

**Figure 9:**
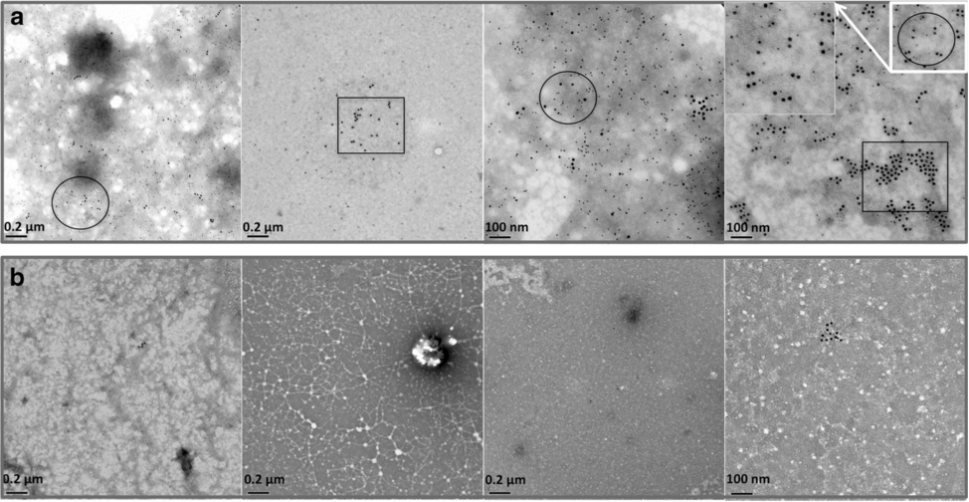
**Immunogold labeling TEM/negatively stain of cerebrospinal fluid from AD brains and age matched controls. a)** A higher density of dityrosine (labeled with 15 nm) and Aβ (labeled with 5 nm) labeling was observed for CSF from an AD patient (top images) compared to **b)** age matched control. In CSF of AD patients, we can identify two different areas of labeling, low-density co-localization (☐), and high density co-localization (◯) of dityrosine and Aβ. We observe significant labeling levels for AD CSF compared to virtually no labeling in CSF from control patients. Inserts show magnified images for further clarity.

Here, TEM immunogold labeling for dityrosine was used to detect dityrosine and gain a general view of protein oxidation represented by dityrosine in CSF of AD and healthy age-matched control subjects. TEM immunogold colabeling/negative staining of CSF reveals a higher density of dityrosine and Aβ labeling in CSF taken from three AD patients (Table 1) (Figure 9a) compared to age matched controls (Table 1) (Figure 9b). Overall there was a much higher density of labeling for both Aβ and dityrosine in AD CSF compared to controls, highlighting the presence of Aβ and also showing the increased level of dityrosine crosslinked proteins in AD CSF. In the CSF from AD patients, two different areas of labeling were observed: those with low-density co-localization, and others showing high-density co-localisation of dityrosine and Aβ indicating that the Aβ found in CSF may contain dityrosine crosslinks, but also that other tyrosine containing proteins are cross-linked in AD CSF.

## Discussion

**Figure 10:**
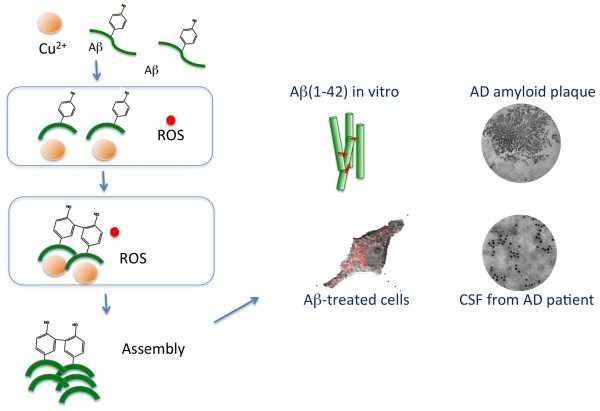
Schematic summarizing the mechanism of dityrosine formation in Aβ in complex with copper including the generation of ROS and summarizing the results showing the physiological importance of dityrosine crosslinked Aβ in Alzheimer’s disease.

Our results have shown that dityrosine crosslinks form in preformed Aβ42 fibrils and in oligomeric Aβ incubated under oxidizing conditions in the presence of Cu^2+^ and H_2_O_2_. We have also confirmed that the dityrosine crosslinked Aβ has the capability to assemble further to form amyloid fibrils. It has been shown previously that 1:1 Cu^2+^: Aβ molar ratios and above are the most efficient ratios to form dityrosine cross-links [45], demonstrating that Cu^2+^: Aβ molar ratio is a critical factor in controlling the aggregation state of Aβ42. We have shown that oxidized Aβ forms stabilized dimer and tetramer and these might represent small oligomeric species that are isolated from AD brain [41]. We hypothesise that the brain derived dimer may be stabilized by dityrosine crosslinks [41,42] and work is on going to confirm this. Here we show that fibrils are stabilized by dityrosine crosslinking. Moreover, it has been shown that sub-stoichiometric Aβ42-Cu^2+^ (10:1) complexes can be toxic to cells [46] and recent work has shown that non-aged and aged molar ratios of 1:1 Aβ42-Cu^2+^ are neurotoxic, whilst Aβ42-Cu^2+^ complexes prepared at sub and supra-equimolar ratios were nontoxic to neuronal cells [22,45]. Aβ peptide can coordinate with Cu^2+^ to give Aβ-Cu^2+^ complexes [47,48], which are redox-active. In turn, these complexes show ability to induce ROS formation, resulting in oxidative stress [16,22]. Cu^2+^ ions coordinate to Aβ via the three histidine residues His6, His13, and His14 to form a histidine bridge [17,49-51] and produce SDS-resistant oligomers [20]. Recent work has shown that Tyr10 is not directly bound to Cu^2+^[51] but that the tyrosine cross-linking is promoted by the histidine bridge formation with Cu^2+^[22]. Taken together, we suggest that dityrosine crosslinking of Aβ could be nucleus for assembly and aggregation to form toxic oligomeric species and amyloid fibrils (Figure 10). An alternative route to dityrosine formation is via myeloperoxidase [52], a peroxidase shown to be associated with Aβ in senile plaques in AD brain tissue [53].

Here, cell culture experiments show that conditions amenable to Aβ dityrosine crosslinking are also present in a cellular environment and that dityrosine crosslinked Aβ fibrils are found around cells and internalized into cells. Aβ and dityrosine co-localize within lysosomes. In previous work, we have shown that internalized oligomeric Aβ42 leads to accumulation of autophagosomes containing Aβ. Considerable evidence supports the view that intralysosomal Aβ accumulation can induce neuronal death [54-56] and here we have shown that oligomers and fibrils can be stabilized by dityrosine crosslinking. Studies using Aβ42 with the tyrosine 10 substituted for alanine mutant showed significantly reduced H_2_O_2_ production and prevented toxicity to primary cortical neurons supporting the view that dityrosine crosslinking may be important in mediating oligomer toxicity [57]. Here, electron micrographs show the internalization of dityrosine crosslinked Aβ fibrillar material into the cytoplasm (Figure 7) and into lysosomes (Figure 6). The oligomeric Aβ may be stabilized by covalent crosslinks and provide a stable nucleus for assembly (Figure 10).

Oxidative stress has been widely implicated in AD pathogenesis. Sources of ROS, such as H_2_O_2_, superoxide anion, and hydroxyl radical, can be formed from different *in vivo* sources (e.g. trace metals, photochemical, and enzymatic reactions) although mitochondria represent the main *in vivo* source of ROS formation [58]. At the same time mitochondria are the main target of ROS attack [59], resulting in the formation of peroxidized, undegradable macromolecules. H_2_O_2_ can easily diffuse into lysosomes and react with iron ions that are released from the degradation of different metalloproteins during their intralysosomal degradation. The interaction of H_2_O_2_ with iron ions results in the formation of the highly reactive hydroxyl radicals. The latter would attack intralysosomal macromolecules, such as Aβ, causing cross-linking of these materials. The oxidative modification especially cross-linking of autophagocytosed material, is the most probable cause of non-degradability of these materials. The excessive accumulation of these non-digested materials could result in endosomal/lysosomal leakage and as a consequent acid hydrolase enzymes will be released and ultimately leading to cell death [56]. Recently, Murakami and Shimizu have clarified the role of cytoplasmic superoxide radical as a possible contributing factor to intracellular Aβ oligomerization in AD [60]. It also has been suggested that intraneuronal Aβ oligomers cause neuronal death by activating endoplasmic reticulum stress, endosomal/lysosomal leakage and mitochondria dysfunction [61,62]. Kurz et al., (2008) [63] have highlighted a close relation between lysosomes and mitochondria, explaining that accumulation of iron inside mitochondria will result in lysosomal iron loading as a consequence of degradation of mitochondria by lysosomal enzymes. Moreover, normal production of H_2_O_2_ by mitochondria results in oxidative stress, which will labilize lysosomes, and further oxidative stress could result from degradation of mitochondria by lysosomal enzymes.

We have shown that amyloid plaques in AD brain tissue show extensive dityrosine crosslinking and this may suggest that these highly stable insoluble deposits are stabilized by the covalent crosslinking resulting in a resistance to degradation. Therefore, the existence of dityrosine may be relevant in AD pathology. Friedrich et al., (2010) [64] have shown that Aβ internalized to cultured cells can accumulate and assemble resulting in eventual cell death and thus they suggest that the formation of amyloid plaques might arise from accumulated intracellular Aβ. Therefore, there may be a pathway from the lysosomal/autophagsosome accumulation of dityrosine crosslinked Aβ that we have observed in neuroblastoma cells to the eventual deposition as amyloid plaques composed of dityrosine crosslinked Aβ observed here in AD tissue. Previous work has revealed that dimeric (9 kDa) Aβ can be isolated from human AD brain [65] and this may be a crosslinked dimer. In future work, it would be important to fully characterize these dimers.

Clioquinol is a potent Cu/Zn chelator and has been shown to significantly reduce Aβ amyloid deposition in an APP transgenic mouse [66] and has shown some efficacy in human AD subjects [67]. These studies have been taken to suggest that copper plays a very significant role in Aβ deposition in AD and could imply that dityrosine formation can stabilize deposits.

A few studies have previously attempted to quantify dityrosine in cerebrospinal fluid. Techniques including HPLC with electrochemical array detection (HPLC-ECD) or fluorescence detection, and liquid chromatography with triple quadrapole mass spectrometric detection (LC-MS/MS) have been applied to quantify dityrosine concentrations in CSF sample from both healthy and disease affected subjects [5,68,69]. However, the results of these studies have been conflicting, perhaps due to differences in sample handling and preparation before measuring. Quantitative screening of protein glycation, oxidation and nitration adducts in CSF of AD and healthy age-matched subjects has been taken to suggest that dityrosine concentration do not change in AD patients with respect to control subjects [68]. By contrast, earlier studies using electrochemical detection showed that dityrosine concentration was elevated markedly in CSF of AD patients [5]. Studies have suggested that CSF is depleted of Aβ in AD patients, however, careful consideration of the results shows highly variable amounts of Aβ in CSF from patients [70]. Here we clearly show, using a specific antibody against the free N-terminus of Aβ42, that Aβ more abundant within AD CSF relative to the age matched controls.

Our results have revealed very strong evidence for the presence of both Aβ and dityrosine in CSF from AD patients and both Aβ and dityrosine are very rarely observed in CSF from age-matched controls. Although this observation needs to be further substantiated with further AD cases, this points to the possible use as a potential biomarker for AD.

## Conclusions

At equimolar ratio of Cu^2+^ and Aβ42, Cu^2+^ can catalyze tyrosine oxidation by H_2_O_2_, leading to dityrosine cross-link generation, and the latter can cause Aβ misfolding, resulting in Aβ assembly into oligomers and subsequently into amyloid fibres. The dityrosine cross-links stabilize Aβ fibres once formed.

Here we present a comprehensive study of *in vivo* and *in vitro* dityrosine cross-linking associated with Aβ. We have revealed the presence of dityrosine cross-links in amyloid plaques in human AD brain, and also in CSF, and identified significant relationships between dityrosine cross-links and amyloid deposit formation. The *in vitro* and *in vivo* results presented here reveal that oligomeric Aβ can undergo oxidative modification in a cellular environment, resulting in cross-linked Aβ through dityrosine followed by intralysosomal Aβ accumulation that could lead to lysosomal leakage and cell death. Our results show the significant accumulation of dityrosine crosslinked Aβ in amyloid plaques, implying a role in stabilization of these insoluble deposits. We have also shown a potential biomarker for AD in CSF, which contains elevated dityrosine crosslinked proteins as well as elevated dityrosine crosslinked Aβ (Figure 9). Research is ongoing to further characterize the crosslinked Aβ as a biomarker in CSF.

## Competing interest

The authors declare that they have no competing interest.

## Authors’ contributions

YA-H conducted and analyzed the experiments and wrote the paper. TW designed experiments, TW, MS-P, ES, LF, MC, WGB conducted experiments, KLM contributed to data analysis, AA-S contributed to experimental design and writing of the paper, JT conducted experiments and analysis and wrote the paper, LCS managed the research and wrote the paper. All authors read and approved the final manuscript.
